# Graphs based methods for simultaneous smoothing and sharpening

**DOI:** 10.1016/j.mex.2020.100819

**Published:** 2020-02-20

**Authors:** Cristina Pérez-Benito, Cristina Jordán, J. Alberto Conejero, Samuel Morillas

**Affiliations:** a Instituto de Biomecánica de València, Universitat Politècnica de València, E-46022 València, Spain; b Instituto Universitario de Matemática Multidisciplinar, Universitat Politècnica de València, E-46022 València, Spain; c Instituto Universitario de Matemática Pura y Aplicada, Universitat Politècnica de València, E-46022 València, Spain

**Keywords:** Color image processing, Local graphs, Simultaneous smoothing and sharpening

## Abstract

We present two new methods for simultaneous smoothing and sharpening of color images: the GMS^3^ (Graph Method for Simultaneous Smoothing and Sharpening) and the NGMS^3^(Normalized Graph-Method for Simultaneous Smoothing and Sharpening). They are based on analyzing the structure of local graphs computed at every pixel using their respective neighbors. On the one hand, we define a kernel-based filter for smoothing each pixel with the pixels associated to nodes in its same connected component. On the other hand, we modify each pixel by increasing their differences with respect to the pixels in the other connected components of those local graphs. Our approach is shown to be competitive with respect to other state-of-the-art methods that simultaneously manage both processes.

• We provide two methods that carry out the process of smoothing and sharpening simultaneously.
• The methods are based on the analysis of the structure of a local graph defined from the differences in the RGB space among the pixels in a 3 × 3 window.
• The parameters of the method are adjusted using both observers opinion and the well-known reference image quality assessment BRISQUE (Blind/Referenceless images spatial quality Evaluator) score.

## Graphical abstract

**Figure:**
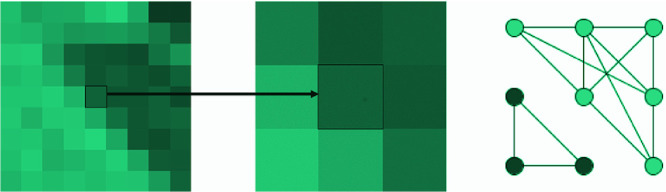

**Table:** Specifications Table

Subject area:	Select one of the following subject areas:
	● Agricultural and Biological Sciences
	● Biochemistry, Genetics and Molecular Biology
	● Chemical Engineering
	● Chemistry
	● Computer Science
	● Earth and Planetary Sciences
	● Energy
	● Engineering
	● Environmental Science
	● Immunology and Microbiology
	● Materials Science
	● Mathematics
	● Medicine and Dentistry
	● Neuroscience
	● Pharmacology, Toxicology and Pharmaceutical Science
	● Physics and Astronomy
	● Psychology
	● Social Sciences
	● Veterinary Science and Veterinary Medicine
More specific subject area:	Image processing
Method name:	Graph Method for Simultaneous Smoothing and Sharpening (GMS^3^)
	Normalized Graph Method for Simultaneous Smoothing and Sharpening (NGMS^3^)
Name and reference of original method:	Cristina Pérez-Benito, Cristina Jordán, Samuel Morillas, and J. Alberto Conejero. A model based on local graphs for color images and its application for Gaussian noise smoothing. J. Comp. Appl. Math. 330, 955–964 (2018).
	Cristina Pérez-Bebito, Cristina Jordán, J. Alberto Conejero, and Samuel Morillas. Graph-based method for simultaneous smoothing and sharpening of color images. Preprint, 2018. J. Comp. Appl. Math. 350, 380-395 (2019).
Resource availability:	The implementation of our methods in Matlab is provided.
	The following functions are needed to run the methods
	fila.m https://pastebin.com/ezr5dgGh clases.m https://pastebin.com/Hjwew3cX

## Introduction

The acquisition of color images is always carried out under non-optimal conditions. Sometimes this is done under low light, too much clarity or poor weather conditions. Also, deficient quality equipment can hamper image acquisition. These conditioners do not only affect the visual perception of the image. They also hinder the identification and distinction of image features that are relevant for different applications such as segmentation or pattern recognition.

To overcome these problems some processes are carried out: on the one hand, an image can be smoothed in order to remove the noise, which is usually of Gaussian type, without losing much image information. On the other hand, for enhancing image details, a sharpening of the borders and details of the picture can be conducted. But even in this last case, smoothing will be needed in order to obtain a robust solution.

Therefore, a combination of both processes can provide optimal results. However, this is not a simple task given the opposite nature of these two operations. The first natural way to proceed is to consider this as a two-step process: first smoothing and later sharpening, or the other way around. However, this approach usually leads to many problems. On the one hand, if we first apply a smoothing technique, then we could be losing information that cannot be recovered in the succeeding sharpening step. On the other hand, if we first apply a sharpening method over a noisy image, we will amplify the noise present in it. The ideal way to address this problem is to consider a method that was able to sharp image details and edges while removing noise.

Many methods for both sharpening and smoothing have been proposed in the literature, but if we restrict ourselves to methods that consider both of them simultaneously, the state-of-the-art is not so extensive. A recent review of the simultaneous application of these methods can be found in [6].

In [7] we have recently proposed the use of local graphs for image smoothing. For any arbitrary pixel, we consider a weighted graph according to the similarities of the pixels in a 3 × 3 window, where pixels in the window stand for nodes, and two nodes are connected if the distance in the RGB space color is smaller than a given threshold U. The cardinal of the set of links of the connected component that contains the central pixel was shown to permit us to separate flat regions from texture and detail regions. If the goal is just smoothing, the threshold *U* can be estimated through maximization of mutual information [3,11]. Later, we have taken this approach for defining two methods that simultaneously address the smoothing and sharpening problems.

## Method details

First, let us describe the steps in which the GMS^3^ can be divided. The NGMS^3^ can be later deduced with a slight modification of the GMS^3^. Given a color image **F**, we consider each image pixel represented by their three color components in the RGB space $F_{0}=\left(F_{0}^{R},F_{0}^{G},F_{0}^{B}\right)$. Except the computations required in Step 0.1, the rest of steps are included in the file GMS3.m.

STEP 0: Definition of the local graph at every pixel.

Consider a 3 × 3 window centered at *F*_0_**.** The rest of the pixels are denoted as $F_{i}=1,\;\ldots ,\;8$.

0.1 The threshold *U* is estimated with ThresholdEstimation.m, which computes the optimal threshold from an estimation of the noise appearing in the image (GaussianNoiseEstimation.m), that, through a regression returns the optimal value (Regression.m). The threshold U is given by the expression U=4.59τ+11.16, where *τ* is an estimation of the standard deviation of the noise. Further details on how this estimation is achieved through a Mutual Information analysis can be found in [7].
0.2 Now, we define the local weighted graph $G_{F_{0}}=\left(V\left(G_{F_{0}}\right),\;L\left(G_{F_{0}}\right)\right)$ for every pixel *F*_0_, where $V(G_{F_{0}})$ stands for the set of nodes and $L(G_{F_{0}})$ for the set of links or edges. Then
  $$V\left(G_{F_{0}}\right)=\left\{F_{i},\;i=0,\;\ldots ,\;8\right\}\;and\;L\left(G_{F_{0}}\right)=\left\{\;\left(F_{i},\;F_{j}\right),\;i\neq j,\;∥F_{i}-F_{j}{∥}_{2}\leq U\;\right\},$$
  with ∥ ∥_2_ standing for the Euclidean norm. Lastly, if $\left(F_{i},\;F_{j}\right)\in L\left(G_{F_{0}}\right)$**,** its weight will be defined as $w\left(F_{i},\;F_{j}\right)=\;∥F_{i}-F_{j}{∥}_{2}$_._

STEP 1: Determination of the connected components of the local graphs.

For this part, we will only consider the pixels whose nodes lay in the same connected component as the pixel *F*_0_. To determine these pixels, we compute the adjacency matrix (AdjacencyMatrix.m) having into account the weights of the links. Then, the connected components are computed with the functions fila.m and clases.m.

STEP 2: Smoothing

We define a kernel-based filter for smoothing, which gives more importance to the pixels closest to the central pixel *F*_0_. The new value for the pixel $F_{0}^{S}$, namely $F_{0}^{S}$**,** will be defined as:

$$F_{0}^{S}:=\frac{\sum_{i\in V\left(CC_{F0}\right)}e^{-\;\frac{{\left|\left|F_{i}-F_{0}\right|\right|}_{2}}{2\alpha^{2}}}F_{i}}{\sum_{i\in V\left(CC_{F0}\right)}e^{-\;\frac{{\left|\left|F_{i}-F_{0}\right|\right|}_{2}}{2\alpha^{2}}}},$$

where *α*  > 0 is a parameter that controls the smoothing effect and *CC*_*F*0_ denotes the connected component that contains the pixel *F*_0_. We will discuss later the fitting of this parameter.

STEP 3: Sharpening

Afterwards, the sharpening part is done taking into account the pixels outside the connected component to which *F*_0_ belongs.

3.1. For the GMS^3^, the new value of the pixel *F*_0_ including the smoothing and sharpening effect will be defined as

$$F_{0}^{GMS^{3}}:=F_{0}^{S}-\lambda v\;being\;v:=\frac{\sum_{i\notin V\left(CC_{F0}\right)}\left(F_{i}-F_{0}^{S}\right)}{9-card\;\left(V\left(CC_{F0}\right)\right)},$$

where λ∈[0,1] is a parameter controlling the sharpening effect, and *card* (*V*(*CC*_*F*0_)) stands for the cardinal of the connected component of the pixel *F*_0_.

3.2. For the NGMS^3^, in order to define the new value of the pixel $F_{0}^{NGMS^{3}}$, we only have to normalize the vector v in the sharpening part.

$$F_{0}^{NGMS^{3}}:=F_{0}^{S}-\lambda \frac{v}{{\left|\left|v\right|\right|}_{2}}$$

The script GMS3.m allows us to execute both methods, thanks to the variable “version”. When calling to GMS3(image, alpha, lambda, version), if version=0 we have the GMS^3^ method, and if version=1 we have the NGMS^3^.

*Implementation:* Both methods, GMS^3^ and NGMS^3^ have been implemented in Matlab.^1^ The implementation can be found in the supplementary material. For running any of these methods we only have to introduce the noisy image, and the values of α and λ. They are implemented in GMS3.m

As it is indicated in [8], the values of *α* and *λ* have been estimated in two different ways:

• From the opinions of a pool of observers. All the observers have visualized the set of images, randomly ordered, under the same conditions: in a dark room, with the same screen, at a distance of about 50 cm of it, and after five minutes of visual adaptation. In this case the optimum parameters of (*α,λ*), where (4.43,0.16) for the GMS^3^ and (8.67,4.54) for the NGMS^3^.
• By minimizing the sum of the squares of the BRISQUE score [4,5] over a dataset of images, using the Interior Point Algorithm [1]. In this case the optimum parameters of (*α,λ*), where (7,0.275) for the GMS^3^ and (5.5,3.5) for the NGMS^3^.

There are differences in the adjustment of the parameters. In the case of the GMS3, the observers smooth and sharpen less than what BRISQUE score would have suggested. However, the opposite effect happens with the NGMS3.

Our methods have been compared with the (i) Forward-and-backward diffusion method (FAB) [9], (ii) the Fuzzy networks based technique (Fuzzy) [10] and, (iii) the collaborative filtering based method (BM3D) [2]. The comparison was based on the well-known non-reference image quality assessment (BRISQUE) score [4,5]. Our methods are competitive with them, both in terms of objective assessment and visual evaluation. Details on this comparison can be found in [8].

## Appendix Supplementary materials

**Supplementary material *and/or* Additional information:***We attach the implementation in Matlab of both methods.* Image, application 1

## Footnotes

1 For the implementation, the following files were used: *fila.m* from https://pastebin.com/ezr5dgGh and clases.m from https://pastebin.com/Hjwew3cX (last access, January 30th, 2018).
