# Supplementary figures and images for: Graphs based methods for simultaneous smoothing and sharpening
Source: MethodsX. 2020 Feb 20;7:100819. doi: 10.1016/j.mex.2020.100819 (PMC7078387; doi:10.1016/j.mex.2020.100819)

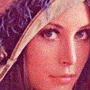

Supplement: Supplementary file 1 — Supplementary material and/or Additional information:We attach the implementation in Matlab of both methods. [file mmc1.zip › lennaDg10.bmp]

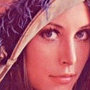

Supplement: Supplementary file 1 — Supplementary material and/or Additional information:We attach the implementation in Matlab of both methods. [file mmc1.zip › lennaDg2p5.bmp]

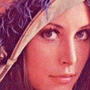

Supplement: Supplementary file 1 — Supplementary material and/or Additional information:We attach the implementation in Matlab of both methods. [file mmc1.zip › lennaDg5.bmp]

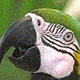

Supplement: Supplementary file 1 — Supplementary material and/or Additional information:We attach the implementation in Matlab of both methods. [file mmc1.zip › parrotsD1g10.bmp]

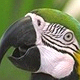

Supplement: Supplementary file 1 — Supplementary material and/or Additional information:We attach the implementation in Matlab of both methods. [file mmc1.zip › parrotsD1g2p5.bmp]

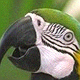

Supplement: Supplementary file 1 — Supplementary material and/or Additional information:We attach the implementation in Matlab of both methods. [file mmc1.zip › parrotsD1g5.bmp]

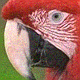

Supplement: Supplementary file 1 — Supplementary material and/or Additional information:We attach the implementation in Matlab of both methods. [file mmc1.zip › parrotsD2g10.bmp]

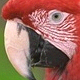

Supplement: Supplementary file 1 — Supplementary material and/or Additional information:We attach the implementation in Matlab of both methods. [file mmc1.zip › parrotsD2g2p5.bmp]

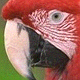

Supplement: Supplementary file 1 — Supplementary material and/or Additional information:We attach the implementation in Matlab of both methods. [file mmc1.zip › parrotsD2g5.bmp]

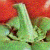

Supplement: Supplementary file 1 — Supplementary material and/or Additional information:We attach the implementation in Matlab of both methods. [file mmc1.zip › peppersDg10.bmp]

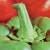

Supplement: Supplementary file 1 — Supplementary material and/or Additional information:We attach the implementation in Matlab of both methods. [file mmc1.zip › peppersDg2p5.bmp]

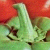

Supplement: Supplementary file 1 — Supplementary material and/or Additional information:We attach the implementation in Matlab of both methods. [file mmc1.zip › peppersDg5.bmp]

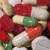

Supplement: Supplementary file 1 — Supplementary material and/or Additional information:We attach the implementation in Matlab of both methods. [file mmc1.zip › pillsDg10.bmp]

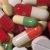

Supplement: Supplementary file 1 — Supplementary material and/or Additional information:We attach the implementation in Matlab of both methods. [file mmc1.zip › pillsDg2p5.bmp]

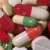

Supplement: Supplementary file 1 — Supplementary material and/or Additional information:We attach the implementation in Matlab of both methods. [file mmc1.zip › pillsDg5.bmp]
